# Gonorrhœal Conjunctivitis

**Published:** 1880-02

**Authors:** Lucien Howe


### Clinical Reports.

---

### GONORRHŒAL CONJUNCTIVITIS — RUPTURE OF CORNEA IN ONE EYE—USE OF TRANSPARENT SHIELD.

BY LUCIEN HOWE, M. D.

Infection of the conjunctiva from gonorrhœal virus is of such frequent occurrence, that any general remarks concerning the disease are necessarily trite and unimportant. In a certain case, however, I have had occasion to make two observations which appear worthy of record, and in order to present these points clearly, it seems well to sketch the outline of the clinical history.

William H., a baker, 19 years old, came to me November 4, 1875, suffering from a severe conjunctivitis of the right eye. He frankly acknowledged the cause of contagion, and stated that his attention was first called to the difficulty in the eye about one week previously.

On examination the lids were found to be œdematous, and swollen to such a degree that no effort on his part would cause them to separate, while a copious discharge of thick and creamy pus continually oozed from between them. On forcibly opening the eye, the conjunctiva was seen to be intensely injected, and so infiltrated that the dull cornea appeared as if half buried. An opaque line extended across that portion, being particularly white and thick at one point, and obstructing the view of the structures beneath. Pain was constant and severe, and vision was already reduced to imperfect perception of light.

With such a condition of affairs, the loss of this eye, either wholly or in part, seemed inevitable, and special precautions were therefore directed to the other, in order, if possible, to protect it from the further contagion which so often results.

For this purpose a shield was fitted to the left, which not only prevented the access to it, of poison from the other side, but at the same time allowed it to be useful for seeing and to be under constant observation. This form of protective bandage was constructed as follows: A thin and clear piece of mica (such as can be found in any hardware store) was cut into a small parallelogram measuring about an inch and a half long by an inch wide. A strip of adhesive plaster was glued to each side of this central transparent window and by then properly bending these, adjusting the ends, and shaping the edges to the brow, cheek and nose, the whole was firmly attached to the parts surrounding the sound eye. Moreover, to guard against any possible openings, the edges and outside of the plaster, especially, where adherent to the skin, were thickly coated with collodion. For reasons which will be mentioned, it was necessary to replace this shield by a similar one several times in the course of treatment, but it will readily be seen that complete and convenient protection was thus afforded to the endangered organ.

The other point in this case which seemed worthy to be noted was the manner of perforation of the corneal ulcer—a process which frequently occurs, but which can seldom be observed. The formation of such an ulcer was apparent from the opacity in the cornea seen at the first examination, and this rapidly increased in size and depth. A strong solution of atropia was of course used frequently, and other attempts made to lessen the corneal inflammation. The conjunctiva was cleansed as frequently and thoroughly as possible, a mild astringent applied occasionally, and once a day a solution of silver nitrate, ten grains to the ounce, was carefully brushed over the inner surface of the lids, being neutralized immediately with salt water.

It was after such an application as this that perforation of the cornea occurred. Special pains had been taken to have the silver solution touch only the inflamed conjunctiva, but a few minutes afterward, when the patient attempted to look upward, suddenly a jet of aqueous humor spurted through the cornea, several inches in front of the face, and great pain was felt in the top and back part of the head.

On examining the eye, the anterior chamber was found to be empty, with iris and lens closely pressed against the cornea; subsequently an extensive leucoma was formed, with the iris adherent to it, a condition which rendered the eye practically useless, although its natural form was retained.

Such are the facts pertaining to these two phases of the case. Let us now consider their practical bearings a little more in detail.

First, as to the protective bandage. That some such precaution is necessary, there seems to be but little doubt among writers, or in the minds of those who have been unfortunate enough to treat many cases of this kind.

Snellen proposed several years ago, that the eye still remaining unaffected, should be covered with a glass coating, but the form here mentioned, seems to be much more simple and convenient than that, and quite as effective as the one described by Graefe or any other.

It is true the frequent bathing of the diseased eye will loosen the adhesive plaster about the nose, and tend to give entrance to the virus unless occasionally renewed.

Moreover, moisture will condense on the inner surface of the mica, not only obscuring the vision in part, but producing a slight degree of conjunctivitis. But these objections are of slight importance compared with the gain, and on the whole this form of the protective bandage would seem to be readily applicable to a large class of cases.

Again, as to the manner and cause of the perforating ulcer. In Zehender's Augenheilkunde (page 189), a case is mentioned in which the same accident occurred quite as suddenly as in the one cited. In that instance he thinks the perforation was produced by forcible contraction of the ocular muscles. It is possible that the same result was due to the same cause in the case which I saw, particularly as the patient was just then making an attempt to look upwards. It is but fair to think, however, that the unfortunate result was at least hastened by the use of the strong solution of silver nitrate.

The fact that such a caustic agent acting on the thin base of the ulcer would be apt to attenuate it still more, the fact that the contact of the solution with the ulcer might happen in spite of the greatest care, and the occurrence of the perforation immediately after the application, all tend to the inference that such might be the possible cause. The use of caustics or even astringents, is of course not indicated at certain stages of a conjunctivitis with a corneal ulcer as a complication, but in such a case, and applied in the manner indicated, there appears to be a large balance of opinion in its favor. I am inclined to think, however, that under circumstances of this sort, strong solutions of any caustic should be used with much caution, if at all, and certainly not until the more acute symptoms have subsided.

